# Exploring the Therapeutic Potential of Natural Products in Polycystic Ovarian Syndrome (PCOS): A Mini-Review of Lipid Profile, Blood Glucose, and Ovarian Histological Improvements

**DOI:** 10.3390/life14010150

**Published:** 2024-01-19

**Authors:** Syawany Wahid, Muhammad Danial Che Ramli, Nur Ezza Fazleen, Rosli Muhammad Naim, Mohd Helmy Mokhtar

**Affiliations:** 1 School of Graduate Studies, Management and Science University, Shah Alam 40100, Malaysia; 012021090471@sgs.msu.edu.my (S.W.);; 2 Faculty of Health and Life Science, Management and Science University, Shah Alam 40100, Malaysia; 3 International Medical School, Management and Science University, Shah Alam 40100, Malaysia; 4 Department of Physiology, Faculty of Medicine, University Kebangsaan Malaysia, Bangi 43600, Malaysia

**Keywords:** natural products, polycystic ovarian syndrome, lipid profile, sex hormone, blood glucose

## Abstract

Polycystic ovary syndrome (PCOS) is a common endocrine disorder in women that is characterized by fluid-filled sacs in the ovaries and various symptoms, including high androgen levels, endometrial irregularities, and cysts. Although the main cause of PCOS remains unknown, it has been linked to genetic, endocrine, and metabolic factors, and there are several treatment options, including lifestyle modifications, medications, and surgery. Natural products such as medicinal plants and fruits are being explored as potential treatments for PCOS because of their bioactive compounds with pharmacological effects related to antioxidant, antimicrobial, anticancer, and antidiabetic properties. Some of these compounds improve insulin sensitivity, reduce inflammation, and enhance glucose metabolism, thereby benefiting patients with PCOS. This mini-review examined the effects of natural products on PCOS, including their effects on ovarian histological changes, blood glucose, sex hormones, and lipid profiles, based on animal and human studies. This study suggests that the use of natural products as complementary medicines can be a promising resource for the development of effective therapeutics for PCOS; however, further research is needed to fully understand their benefits.

## 1. Introduction

Polycystic ovary syndrome (PCOS), also known as Stein–Leventhal syndrome, is the most common endocrine abnormality in women [1]. It is a collection of symptoms that affect the ovaries and ovulation. PCOS is a complex disorder caused by high androgen levels, irregularities in the endometrium, and some cysts in the ovaries. The leading causes of PCOS are genetics, lifestyle, and environmental factors [2]. PCOS is associated with genetic, endocrine, and metabolic factors, but the exact cause remains unknown. In addition, it is associated with multiple symptoms, such as ovarian enlargement [3], abdominal obesity [4], impaired metabolism disorder [5], insulin resistance [5], type 2 diabetes [6], and dyslipidemia [7].

According to the World Health Organization (WHO), there were 1.55 million cases of PCOS in women aged 15–49 years in 2017, an increase of 4.47% (2.86–6.37%) from 2007 [2]. The prevalence of this disease was reported to be 5.6–8% in Europe [8]. According to recent studies, the prevalence of PCOS in Malaysia is 36.23%, based on the Rotterdam criteria, and 6.8%, based on the National Institute of Health (NIH) [9]. It has been shown that the cause of infertility is failure to ovulate in approximately 75% of cases [8]. PCOS has significant implications for achieving the United Nations’ Sustainable Development Goals (SDGs), particularly SDG 3 (good health and well-being) [2].

Various therapies have been used to treat PCOS, such as lifestyle modification, surgical treatment for ovulation induction, and other medications [10]. Oral contraceptives, such as metformin and clomiphene, are the most commonly used treatments for PCOS. Metformin is commonly used in PCOS patients to treat and alleviate the metabolic issues of glucose tolerance and insulin resistance, and it is especially suggested for the PCOS metabolic phenotype [11]. However, these drugs can cause side effects, such as anorexia, diarrhea, and abdominal pain [12]

In recent years, natural products have shown significant phytochemical isolation to improve the condition of PCOS patients [13]. Plants with medicinal value are part of human culture and tradition. They have significant nutritional value and are prescribed for various therapeutic purposes [14]. Over 21,000 plant species reported by the WHO have therapeutic value and can be used as medicines [15]. Plants, animals, fungi, and microorganisms can form natural products [16]. In this study, we reviewed the effects of natural products on PCOS, including ovarian histological alterations in both animal and human studies, as well as blood glucose, sex hormones, and lipid profiles.

## 2. Methods

The present study examined a mini-review of relevant English-language publications published on PCOS, herbs, medicinal herbs, and plants. International databases such as PubMed, Science Direct, Frontiers, Scopus, and ISI repositories from 2010 to 2023 were searched to identify animal studies and clinical trials on herbal treatments for PCOS. The current search was conducted using medical terminology terms and combinations of keywords using the following search strategy: “polycystic ovary syndrome” or “PCOS” and “natural plant” or “herb” or “ovarian cysts” or “fruits” or “medicinal herbs”. The references cited in each article found in the automated search were also examined. Non-English or irrelevant articles were not included in the review. Reviews, case reports, short communications, and letters to the editor were also not included in the study.

## 3. Literature Review

### 3.1. Natural Products

Natural products have recently gained popularity because of their potential therapeutic benefits and minimal adverse effects. Phytochemicals extracted from medicinal herbs and plants represent a crucial avenue for exploring and identifying innovative pharmaceutical agents [17]. They have been used for centuries for medicinal purposes [16]. Many medicinal plants have been reported to exhibit several pharmacological effects related to vital functions, including antioxidant, antimicrobial, anticancer, and antidiabetic properties [14]. Because plants serve as repositories for various phytochemicals, they can be used to treat various diseases. These chemicals include alkaloids, flavonoids, terpenoids, phenolic acids, and other compounds [14]. Each of these compounds has a unique chemical composition and mode of action, which may explain their various medicinal benefits [16].

Alkaloids are a class of nitrogen-containing plant chemicals with many biological functions [18]. Nevertheless, based on the existing body of literature, alkaloids exhibit limited promise in the treatment of PCOS. *Berberis darwinii Hook* [19], *Berberis aquifolium Pursh*. [20], and *Berberis aristata DC.* [21] have improved anti-inflammatory, anticancer, and antidiabetic effects in patients with PCOS [22].

Another compound in natural herbs is quercetin (3,5,7,3′,4′-pentahydroxyflavone), a dietary flavonoid found in numerous fruits and vegetables. It has garnered significant attention owing to its antioxidative, anti-inflammatory, antitumor, hypoglycemic, cardiovascular protective, and other properties in regulating ovarian function and preserving ovarian morphology [23,24,25]. The research revealed that quercetin, which serves as the primary constituent of the Kuntai capsule, a proprietary Chinese medicine, exhibits a significant overlap with potential therapeutic targets for treating polycystic ovary syndrome (PCOS) [26].

Terpenoids are broad and varied bioactive compounds present in numerous plants, fungi, and mammals [27]. Terpenoids, including ginsenosides, have anti-inflammatory, antioxidant, antiobesity, and antidiabetic properties [27]. Limonene, found in citrus fruits, has strong potential antioxidant properties and has been shown to improve lipid metabolism and reduce inflammation [28,29]. Many seeds, fruits, vegetables, and cereals contain a group of substances known as phenolic acids. They are known for their anti-inflammatory and antioxidant effects [30,31]. Some of these have been shown to help women with PCOS improve insulin sensitivity and reduce insulin resistance [31]. For example, studies in women with PCOS have shown that chlorogenic acid in coffee and many fruits reduces insulin resistance and enhances glucose metabolism [32]. Several other bioactive chemicals discovered in natural products have also shown beneficial effects on PCOS symptoms. These include omega-3 fatty acids in fish and nuts and resveratrol in red grapes and peanuts [33].

Flavonoids are a significant class of biologically active compounds commonly present in nature. They exhibit a diverse range of physiological effects, including anti-inflammatory properties [34], antioxidative activity [35], hypoglycemic effects [36], antiviral properties [37], and antitumor activity [36].

Overall, natural products offer a variety of bioactive substances that could be therapeutically useful in PCOS-affected women. Natural products represent a viable alternative or complementary therapy for PCOS treatment, although further studies are needed to fully understand their benefits. Table 1 summarizes the characteristics, lipid profiles, sex hormones, blood glucose levels, and histological changes.

### 3.2. Lipid Profile Improvement

All aspects of the lipid profile could be affected if the patient has PCOS. Several studies have shown the potential of natural products to improve lipid profiles associated with PCOS [54]. Dyslipidemia is a condition characterized by abnormal levels of lipids in the blood. It is often characterized by high triglyceride (TG) levels and low high-density lipoprotein (HDL) levels. These abnormal lipid levels can increase the risk of cardiovascular disease, stroke, and other health problems [55]. Dyslipidemia is not a main diagnostic criterion but a significant metabolic abnormality [56]. It has been discovered that low-density lipoprotein (LDL), TGs, and HDL have a 70% prevalence in PCOS patients [54]. Dyslipidemia in PCOS is related to hyperandrogenism, in which androgens decrease the catabolic clearance of LDL by interacting with the androgen receptor (AR) [57]. Dyslipidemia was also found to impact PCOS patients’ long-term outcomes.

#### 3.2.1. Low-Density Lipoprotein (LDL)

Previous studies have shown that LDL levels are elevated in women with PCOS, which is unusual in insulin-resistant conditions. Although the cause of elevated LDL levels in women with PCOS is unknown, they may be linked to hyperandrogenism or genetic components [54]. According to the review, several studies show significant reductions in LDL levels [40,45,51,58]. The reduction in LDL levels has been attributed to the presence of polyphenols. Resveratrol is an example of a polyphenol that may improve lipid metabolism and LDL cholesterol levels. These effects can be attributed to their antioxidant and anti-inflammatory properties [16,56]. Studies have shown that natural products contain flavonoids, among the phytochemicals found in natural plants, which have demonstrated the ability to lower LDL cholesterol levels [54], while quercetin, catechins, and anthocyanins are flavonoids linked to potential cardiovascular advantages, such as lowering LDL cholesterol levels [42,59]. Flavonoids may have an effect by lowering LDL oxidation, enhancing endothelial function, and modifying lipid metabolism. These phytochemicals can lower LDL cholesterol levels through several mechanisms, including lowering LDL oxidation, increasing LDL receptor activity, inhibiting cholesterol synthesis, and altering lipid metabolism [60].

Three studies reported unchanged LDL levels [44,50,53]. PCOS is characterized by hormonal imbalances, including elevated levels of androgens, such as testosterone. While these hormonal imbalances can affect lipid metabolism and contribute to changes in high-density lipoprotein (HDL) cholesterol levels, they may have a lesser impact on LDL cholesterol levels [61]. Insulin resistance is a common feature of PCOS, in which the body has difficulty effectively utilizing insulin. Insulin resistance can lead to dyslipidemia, an abnormal lipid profile characterized by elevated triglyceride and decreased HDL cholesterol levels. However, its impact on LDL cholesterol may be less pronounced [7].

#### 3.2.2. High-Density Lipoprotein (HDL)

Studies have shown that PCOS increases the HDL levels in the blood serum [25,28], while several studies have shown the opposite result [44,45,49,58]. This condition occurs because of hormonal imbalances and insulin resistance. PCOS is characterized by hormonal imbalances, including elevated levels of androgens, such as testosterone. It has been suggested that increased androgen levels can stimulate the liver to produce more HDL cholesterol [62]. Insulin resistance is a common feature of PCOS, in which the body has difficulty effectively utilizing insulin. Insulin resistance can lead to dyslipidemia, an abnormal lipid profile characterized by elevated triglyceride levels and decreased HDL cholesterol [63]. However, HDL levels can increase in some PCOS patients, potentially due to compensatory mechanisms or other factors associated with the condition. Olive oil, which includes extra virgin olive oil in the diet, has been shown to positively affect lipid profiles, including increasing HDL cholesterol levels. Olive oil is a monounsaturated fat that can be used as an alternative to saturated and trans fats [45]. Obesity and a sedentary lifestyle are common in PCOS and can further contribute to dyslipidemia, including decreased HDL cholesterol levels [64]. Lack of physical activity and excess bodyweight can negatively affect lipid levels [65].

Based on this review, studies have shown that the HDL level remains unchanged. Genetic factors can influence HDL cholesterol levels [5,66]. Some individuals may have a genetic predisposition to high or low HDL cholesterol levels, which may remain relatively stable despite lifestyle changes or treatments. Flavonoids and resveratrol have the potential to improve HDL cholesterol levels.

#### 3.2.3. Triglycerides (TGs)

Several studies have shown a significant decrease in the TG levels in PCOS patients [40,44,45,58]. Catechins and flavonoids have been linked to a potential reduction in triglyceride levels and have high antioxidant and anti-inflammatory effects [27,59,67]. Moreover, resveratrol is associated with improved lipid profiles and may help to lower TG levels [49]. Studies have shown an increase in TG levels after treatment with natural products [49,50]. Insulin resistance in PCOS often leads to hyperinsulinemia. Insulin plays a role in lipid metabolism and stimulates the liver to produce more triglycerides [63]. High insulin levels also decrease the breakdown of TGs in fat tissue, further contributing to elevated triglyceride levels. PCOS is characterized by imbalances in female sex hormones, including elevated levels of androgens, such as testosterone. These hormonal imbalances can affect lipid metabolism and increase TG levels [7,61].

In summary, natural products can potentially improve the lipid profiles of women with PCOS through their antihyperlipidemic effects, glucose metabolism enhancement, enhancement of absorption, and alteration of serum biochemical variables related to lipolysis [10]. Further research is needed to fully understand the mechanisms of action of natural products and determine their optimal dosage and treatment duration for patients with PCOS.

### 3.3. Blood Glucose Improvement

Blood glucose levels are a significant symptom commonly observed in PCOS patients [54]. Consequently, pharmacological agents that enhance insulin sensitivity have been incorporated into therapeutic regimens for polycystic ovary syndrome [68]. It has been proposed that PCOS is a condition characterized by oxidative stress [69]. The antioxidant function of the human body is insufficient to effectively manage the excessive presence of reactive oxygen species (ROS) [70]. This inadequacy contributes to the worsening of the clinicopathological characteristics associated with PCOS in patients, including chronic oligo-anovulation or anovulation, clinical or biochemical indications of hyperandrogenism, and the presence of polycystic ovarian morphology. In the interim, elevated levels of oxidative stress exacerbate physiological responses and frequently coincide with insulin resistance.

Several studies have shown significant improvements in blood glucose levels after treatment with natural products [41,42,43,50,51,52,58]. The improvement in blood glucose levels showed a decreased tolerance to glucose. Most natural products may increase glucose tolerance, and they have phytochemical properties, such as thylakoid, flavonoid, and phenolic compounds, which can affect the secretion and metabolism of insulin and its action [31,59].

Based on previous studies, cinnamon and berberine contain phenolic compounds. Cinnamon is a spice from the genus Cinnamomum that contains phenolic compounds that may improve insulin sensitivity and glucose uptake and reduce fasting blood glucose levels in individuals with PCOS [44,52]. Berberine, found in plants such as barberries, has potential antidiabetic effects. Flavonoids can be found in berries and olive oil, which act as antidiabetic agents, improve glucose metabolism and insulin sensitivity, and reduce inflammation. These antidiabetic effects of natural products help manage diabetes in patients with PCOS [71].

In addition, natural plant products can decrease glucose resistance by increasing insulin levels and regulating glucose production. Therefore, natural products help reduce glucose resistance by controlling glucose homeostasis, improving insulin secretion, and enhancing insulin-mediated glucose uptake [42]. Impaired glucose metabolism is highly prevalent in women with PCOS, which can lead to diabetes mellitus type 2 [12]. Several studies have reported no significant differences after treatment [19,20]. The abnormal production of blood glucose in patients with PCOS has been associated with deficient insulin secretion. Rosklint et al. reported that high androgen production can cause insulin resistance [72]. According to previous research, androgen may contribute to insulin resistance and alter how insulin functions in the target tissue in PCOS [72].

Although investigations into the therapeutic efficacy of natural products for managing polycystic ovary syndrome (PCOS) and enhancing blood glucose levels are still underway, preliminary results indicate their potential to ameliorate metabolic irregularities linked to the disorder. Nevertheless, additional meticulously planned clinical trials are necessary to establish the effectiveness of these organic substances, their ideal dosage, and their enduring impact.

### 3.4. Sex Hormone Improvement

Measurement of sex hormone levels, especially testosterone and luteinizing hormone (LH) levels, is recommended for PCOS diagnosis [17]. Low levels of progesterone and follicle-stimulating hormone (FSH) and increased serum levels of LH and testosterone are the most reliable indicators of PCOS in women [22]. The induction of PCOS and treatment with various extracts have been shown to decrease testosterone and LH levels and increase progesterone and FSH levels [23].

#### 3.4.1. Testosterone

Several studies have reported elevated serum testosterone levels [40,43,44,51,52]. PCOS is characterized by elevated serum testosterone levels, which is known as hyperandrogenism. The multifactorial causes involve hormonal imbalances, insulin resistance, hyperinsulinemia, and elevated LH levels [61]. Ovarian dysfunction disrupts normal ovulation, leading to excessive androgen production. Insulin resistance causes cells to become less responsive to insulin, resulting in higher blood levels [12,61]. Hyperinsulinemia stimulates the ovaries to produce androgens, including testosterone [73]. Elevated LH levels contribute to increased androgen production in ovaries [40,45]. Examples include spearmint tea and flaxseeds. Spearmint tea has been investigated for its potential antiandrogenic effects, whereas flaxseed contains lignans, which have been studied for their potential hormonal effects [46].

Several studies reported that the testosterone level increased after receiving treatment [38,39,41,47,48]. Tumors have the potential to overproduce androgens, resulting in increased testosterone levels. Supplements and drugs can also directly increase serum testosterone levels. Hormone imbalances can cause symptoms, such as hirsutism, acne, and irregular menstrual cycles [61,62,68].

#### 3.4.2. Progesterone

Progesterone is primarily produced by the ovaries in females and, to a lesser extent, by the adrenal glands in both males and females. Progesterone, the synthesis of which depends on the corpus luteum, regulates reproductive cycles and assists the uterus in implantation during conception [74]. Progesterone levels significantly increased after treatment in several studies [39,40,41,46], and significantly decreased in another study [50,51]. Phytoestrogens contain compounds that mimic or interact with estrogen in the body. Plants that contain isoflavones have estrogenic effects, which can help increase progesterone levels in PCOS patients [16,31].

#### 3.4.3. Luteinizing Hormone (LH)

Polycystic ovary syndrome (PCOS) is an endocrine disorder characterized by increased luteinizing hormone (LH) levels. The cause is unclear, but it is believed to be related to hormonal imbalances and disrupted feedback mechanisms in the reproductive system [12]. Factors contributing to increased LH levels include excessive androgen production, insulin resistance, dysregulated gonadotropin-releasing hormone (GnRH) levels, and disrupted feedback mechanisms. These factors can lead to anovulation, menstrual irregularities, and increased androgen production [75]. Not all women with PCOS experience the same degree of LH elevation, and individual symptoms and hormonal profiles can vary [12,61,75]. Studies have reported that LH levels significantly decrease after natural treatment [32,39,40,41,50,51,58]. Phytoestrogens are plant compounds that have estrogenic effects. Some phytoestrogens, such as genistein and daidzein, found in soy and other legumes, may have mild estrogenic activity and interact with estrogen receptors in the body. Although their effects on LH are not well established, phytoestrogens can influence the hormonal balance and feedback mechanisms [16,31].

#### 3.4.4. Follicle-Stimulating Hormone (FSH)

FSH levels have been shown to increase in several in vivo and clinical studies in patients with PCOS [32,38,39]. Polycystic ovary syndrome (PCOS) affects follicle-stimulating hormone (FSH), a hormone that is crucial for ovarian follicle growth and development. Disrupted feedback mechanisms, insulin resistance, and dysregulated GnRH levels can lead to increased FSH levels [12,61,75]. Hormonal imbalances in PCOS vary among individuals, and the exact mechanisms underlying these increases are still under investigation. Imbalances in FSH levels contribute to the characteristic features of PCOS, such as irregular menstrual cycles, anovulation, and ovarian cyst formation [61].

Several studies have reported unchanged FSH levels after treatment with natural products [51,58]. A complex interplay of hormones influences FSH levels, and elevated FSH levels can result from hormonal imbalances in the body [12]. If the treatment primarily focuses on addressing other hormonal imbalances, such as reducing luteinizing hormone (LH) levels or insulin resistance, then it may not directly affect FSH levels. In polycystic ovarian syndrome, abnormal steroidogenesis is manifested by an increase in the production of androgen and estradiol, and the malfunctioning hypothalamic–pituitary–ovarian axis is manifested by an increase in the secretion of LH and GnRH and a reduction in the FSH concentration [68].

### 3.5. Histological Change Improvement

Plant-based or herbal natural products have demonstrated potential for mitigating histological alterations associated with PCOS. Several studies have concentrated on hormonal interventions and developmental stages that result in changes in ovarian morphology, most commonly an increase in the numbers of antral and cystic follicles, and a decrease in the corpus luteum. An increase in the progesterone level is associated with reduced corpus luteum production [38,76]. Ndeingang et al. (2019) reported that the numbers of cystic and antral follicles decreased and increased, respectively, in the corpus luteum after receiving treatment [51]. Studies have reported that the number of cysts decreases and the number of antral follicles increases in the corpus luteum after treatment [39,40,42,43,46,47,49,50,51,52,58]. Natural product treatments can affect the numbers of ovarian cysts and antral follicles, as well as the corpus luteum (CL). Enhanced corpus luteum formation, a temporary structure that develops from the ruptured follicle after ovulation, is crucial for maintaining the uterine lining for a potential pregnancy [59,62,63]. These treatments can help regulate hormone levels, improve insulin sensitivity, reduce inflammation, and improve ovarian function.

Kakadia et al. (2019) reported that ovarian histology in the treatment group showed normal follicular development and decreased cyst formation. Gharanjik et al. (2022) and Sherafatmanesh et al. (2020) reported that the corpus luteum increased and the antral follicles did not change significantly. The number of cystic follicles may decrease because of decreased serum testosterone levels [77]. This is because most natural compounds contain flavonoids, which have antioxidant properties and can reduce free radical formation in the ovary [11,60,78]. In general, the conclusions of studies on hormonal interventions and developmental stages at the time of the study varied widely. Most resulted in ovarian morphological changes, mainly an increase in the numbers of antral and cystic follicles, and a decrease in the size of the corpus luteum. Using androgens and natural products as stimulants could produce a hyperandrogenic state.

### 3.6. The Efficacy of Natural Products and Drug Therapy

In addition to being more affordable and having fewer side effects, natural medicines, such as plants and herbs, have long been used to treat several gynecological conditions [16]. Various disorders, including PCOS, have been aided by the use of numerous herbal treatments. Metabolic problems in patients with PCOS have been the focus of their positive effects on PCOS symptoms. Owing to high financial expenditures and many adverse consequences associated with the use of allopathic drugs, the demand for herbal remedies has surged [15]. Table 2 shows a Summary of different treatment options for the management of PCOS.

The antioxidant and anti-inflammatory potential of plant sources has been widely studied for a long time. Most natural products can improve PCOS hormone levels because they contain various phytochemicals [11,59,60]. Based on a review, most natural products have anti-inflammatory and antioxidant properties, which can help with PCOS symptoms [67]. A review by Ashkar et al. and Rababa’h et al. (2020) stated that the combination of resveratrol, barberry, and marjoram has a high level of antioxidant and anti-inflammatory effects to regenerate ovarian morphology and improve PCOS symptoms [22,71]. Sherafatmanesh et al., in the year 2020, also stated that the combination of spinach and caraway has high levels of antioxidants, such as flavonoids, and antiobesity and anti-hyperlipidemia properties [58]. Kakadia et al. and Gharanjik et al. (2022) stated that *Vitex negundo* L. and *Calendula officinalis* have anti-inflammatory and antioxidant properties that have been used in gynecological disorders due to the phenolic compounds in the extracts. *N. sativa* seeds have hypoglycemic and anti-inflammatory properties, which help to improve hormonal levels [41,50]. Strong antioxidant properties protect the body from the damaging effects of free radicals and improve ovarian morphology, thereby alleviating PCOS symptoms.

Elevated androgen levels are also one of the major etiologies of PCOS. Some natural products have antiandrogenic and antiestrogenic properties, which can help improve hormonal levels in patients with PCOS [61,62]. Previous studies have shown that natural products contain a high level of antiandrogenic and antiestrogenic properties, which can improve endocrine secretion, including estradiol, progesterone, and testosterone levels [41,79]. Aliakbari et al., in the year 2022, stated that the combination of *B. persicum* capsules and *F. vulgare* has a phytoestrogen that can help improve hirsutism, BMI, and menstrual duration. Antiandrogens may help the body maintain biosynthesis and release estrogen [79]. Phenolic compounds in natural products are effective against PCOS [30,31]. Most plants have anti-inflammatory and antioxidant properties and show anti-PCOS efficacy [35,55,57].

Drug therapy helps to treat hyperandrogenism, infertility, or other conditions, depending on the identification and management of symptoms. Patients with PCOS who wanted to regain fertility were treated with metformin and clomiphene [10]. Metformin is one of the medicines used in insulin sensitizers for treating PCOS and has long been used to treat type 2 diabetes [10,12]. While other medicines, such as clomiphene, have been used to treat infertility, women with PCOS have been advised to take metformin if they have low lipid profiles, glucose intolerance, or both [59]. Diarrhea, nausea, and gastrointestinal disorders have also been linked to the complex long-term use of both drugs [61]. Patients with PCOS and infertility are frequently advised to use oral contraceptives, the main purpose of which is to regulate menstruation [12]. These medications also lower testosterone levels, which reduces hirsutism and acne. These drugs are more effective at treating androgenic symptoms than the earlier versions. Long-term oral contraceptive use can result in weight gain, nausea, vomiting, and arterial hypertension. Spironolactone and gonadotropins are other treatment options [12,61]. In patients with PCOS, both medications have been used to inhibit androgens. Long-term gonadotropin therapy for women with anovulatory PCOS can result in vaginal dryness, depression, and loss of bone mass. Spironolactone is the most widely used antiandrogen and is safe, readily available, and inexpensive [10]. The negative effects of prolonged use may result in irregular menstrual cycles, hyperkalemia, and hypotension [12,61].

However, drug therapy is more specifically targeted for PCOS symptoms and leads to satisfactory clinical results. Based on this review, drug therapy can also lead to other complications in patients with PCOS. Natural products are safe and effective therapies for the treatment of polycystic ovarian syndrome, preferably combined with improved hormone levels, hirsutism, and insulin resistance.

The therapies for PCOS are not completely effective and have some side effects; thus, it is essential to arrive at more beneficial therapeutic alternatives with fewer risks of side effects. Consequently, therapeutic plants with several active ingredients and few side effects have gained popularity. According to a large number of studies, infertility, hormonal status, insulin resistance, and lipid profiles are just a few of the clinical and laboratory symptoms of PCOS that can be treated with a variety of plants [7,15]. The main mechanisms underlying the effectiveness of medicinal plants in PCOS are not yet fully understood. However, they are highly beneficial in the treatment of PCOS. Thus, large-scale in vivo and in vitro studies are needed to test the potential side effects of medicinal plants on PCOS.

## 4. Conclusions

PCOS is a prevalent endocrine disorder among women from adolescence to premenopause. The condition is associated with various complications, such as infertility, metabolic and cardiovascular disorders, and chronic health issues that may persist throughout an individual’s lifetime. Pharmaceutical interventions have demonstrated the effective management of polycystic ovary syndrome (PCOS); however, significant incidences of adverse drug reactions have raised concerns regarding their potential for long-term curative benefits. Patients are turning to herbal therapy as a substitute for synthetic medications in the management and treatment of PCOS to improve recovery rates and increase acceptance. The present review offers a comprehensive analysis of herbal remedies that confer benefits to PCOS and its associated complications. Plant medications cannot fully replace the conventional treatment in every case. However, they are highly beneficial in PCOS therapy. This review shows that natural products can be an alternative to help patients with PCOS because of the phytochemicals contained in the plants themselves. These natural products can alleviate changes in ovarian histology, blood glucose, serum hormone levels, and lipid profiles. Therefore, natural products as alternative medicines can be considered promising resources for the development of effective therapeutics for PCOS.

## Figures and Tables

**Table 1:** Summary of study characteristics and lipid profiles, sex hormones, blood glucose, and histological changes.

References	Intervention	Time of Experiment and Sample Size	Study Design	Lipid Profile	Sex Hormone	Blood Glucose	Histological Changes
Ibrahim et al., 2022 [38]	Pomegranate juice extract (PJE) (400 mg/kg/day)	3 weeks + 40 rats	Animal	Not measured	FSH: increase LH: increase Testosterone: increase Progesterone: not measured	Not measured	Increase in endometrial collagen content
Aliakbari et al., 2022 [39]	*B. persicum* capsule (60 mg) + *F. vulgare* capsule (25 mg)	4 months + 70 women with PCOS	Human	Not measured	FSH: increase LH: decrease Testosterone: increase Progesterone: increase	Not measured	Corpus luteum: not measured Number of follicles: decrease
Younas et al., 2022 [40]	Ethanolic extract of *Fagonia indica* (500 mg/kg)	7 weeks + 25 female rats	Animal	TG: decrease TC: decrease LDL: decrease HDL: increase	FSH: increase LH: decrease Testosterone: decrease Progesterone: increase	Not measured	Corpus luteum: not measured Number of follicles: decrease
Younas et al., 2022 [40]	Kelulut honey (0.5 g/kg/days, 1 g/kg/day, 2 g/kg/day)	35 days + 24 female rats	Animal	Not measured	Not measured	Not significant	Corpus luteum: decrease Number of follicles: decrease
Gharanjik et al., 2022 [41]	Hydroalcoholic extract of *Calendula officinalis* (200, 500, and 1000 mg/kg)	35 days + 60 female adult rats	Animal	Not measured	FSH: increase LH: decrease Testosterone: increase Progesterone: increase	Decrease	Corpus luteum: decrease Number of follicles: decrease
Peng et al., 2021 [42]	*Eucommia ulmoides Oliv*. leaves (TFEL)	21 days + 60 rats	Animal	Not measured	Not measured	Decrease	Corpus luteum: decrease Number of follicles: decrease
Khani et al., 2021 [43]	Hydroalcoholic extract of N. Sativa seeds (50, 100, and 200 mg/kg)	30 days + 36 rats	Animal	Not measured	FSH: decrease LH: increase Testosterone: decrease Progesterone: not measured	Decrease	Corpus luteum: increase Number of follicles: decrease
Permadi et al., 2021 [44]	*C. burmanii* + *L. spesiosa* extract (100 mg)	+62 volunteers with PCOS	Human	TG: decrease TC: decrease LDL: unchanged HDL: decrease	FSH: not measured LH: not measured Testosterone: decrease Progesterone: not measured	Not measured	Not measured
Yahay et al., 2021 [45]	Canola and olive oil (25 g)	10 weeks + 72 women	Human	TG: decrease TC: decrease LDL: decrease HDL: decrease	Not measured	Not measured	Not measured
Mehraban et al., 2020 [46]	Combination of spearmint extract (SE) and flaxseed extract (FE)	30 days + 24 rats	Animal	Not measured	FSH: not measured LH: not measured Testosterone: increase Progesterone: increase	Not measured	Corpus luteum: decrease Number of follicles: decrease
Mvondo et al., 2020 [47]	Aqueous *M. arboreus* extract (20, 110, and 200 mg/kg)	30 days + 60 adult rats	Animal	Not measured	FSH: not measured LH: increase Testosterone: increase Progesterone: not measured	Not measured	Corpus luteum: increase Number of follicles: decrease
Rababa’h et al., 2020 [48]	Marjoram extract (20 mg/kg)	3 weeks + 75 adult rats	Animal	Not measured	FSH: not measured LH: not measured Testosterone: increase Progesterone: increase	Not measured	Not measured
Ashkar et al., 2020 [49]	Hydroalcoholic extract of *Berberis integerrima* and resveratrol (3 g/kg of barberry and 20 g/kg of resveratrol)	42 days + 70 adult rats	Animal	TG: increase TC: increase LDL: increase HDL: decrease	Not measured	Unchanged	Corpus luteum: increase Number of follicles: decrease
Kakadia et al., 2019 [50]	Thylakoid-rich spinach extract and aqueous extract of caraway (*Carum carvi* L.)	8 weeks + 60 rats	Animal	TG: decrease TC: decrease LDL: decrease HDL: increase	FSH: unchanged LH: decrease Testosterone: not measured Progesterone: not measured	Decrease	Corpus luteum: increase Number of follicles: decrease
Kakadia et al., 2019 [50]	*Vitex negundo* L. extract, (200 and 400 mg/kg)	40 days + 30 rats	Animal	TG: increase TC: unchanged LDL: not measured HDL: unchanged	FSH: decrease LH: decrease Testosterone: increase Progesterone: decrease	Decrease	Corpus luteum: increase Number of follicles: decrease
Ndeingang et al., 2019 [51]	Methanolic extract of *P. muellerianus* (30, 60, 120 mg/kg)	14 days + 114 rats	Animal	TG: unchanged TC: decrease LDL: decrease HDL: increase	FSH: unchanged LH: decrease Testosterone: decrease Progesterone: decrease	Decrease	Corpus luteum: increase Number follicles: decrease
Dou et al., 2018 [52]	Cinnamon powder (10 mg/100 g)	20 days + 50 mice	Animal	Not measured	FSH: increase LH: decrease Testosterone: decrease Progesterone: not measured	Decrease	Corpus luteum: increase Number follicles: decrease
Mannerås et al., 2010 [53]	Malaysian herb *Labisia pumila* var. *Alata* (LPva) extract (50 mg/kg)	5 weeks + 20 rats	Animal	TG: unchanged TC: unchanged LDL: unchanged HDL: unchanged	Not measured	Not measured	Not measured

TC, total cholesterol; TG, triglyceride; HDL, high-density lipoprotein; LDL, low-density lipoprotein; LH, luteinizing hormone; FSH, follicle-stimulating hormone.

**Table 2:** Summary of different treatment options for the management of PCOS.

Drugs	Effects	Adverse Effects
Drug therapy		
Metformin	Reduces insulin sensitivity, improves lipid profile, helps in reducing bodyweight, infertility treatment	Diarrhea, nausea, gastrointestinal disorder
Clomiphene	Infertility treatment	Headache, nausea, abdominal pain
Oral contraceptives	Restore regular periods, reduce symptoms of hyperandrogenism	Nausea, vomiting, bodyweight gain, arterial hypertension
Gonadotropins	Inhibit androgen	Vaginal dryness, depression, loss of bone mass
Spironolactone	Inhibits androgen	Irregular menstrual cycles, hyperkalemia, hypotension
Anti-inflammatory	Inhibits androgen	Irregular menstrual cycles, hyperkalemia, hypotension
Insulin-sensitizing effects	Inhibit androgen	Irregular menstrual cycles, hyperkalemia, hypotension
Natural product		
Pomegranate juice extract	Improves endometrial receptivity and normalizes hormonal level	No side effects
Combination of *B. persicum* and *F. vulgare*	Decreases hirsutism and BMI and increases menstrual duration	No side effects
*Fagonia indica*	Restores ovarian morphology and downregulates serum levels of testosterone	No side effects
*Calendula officinalis*	Restores blood glucose and promotes folliculogenesis in the ovarian tissue	No side effects
Olive leaves	Inhibit ovarian hyperplasia and restore blood glucose	No side effects
*N. sativa* seeds	Improve hormone level in PCOS	No side effects
Combination of *C. burmanii* and *L. spesiosa* extract	Improves testosterone level in PCOS	No side effects
Combination of canola and olive oil	Improves endometrial receptivity and normalizes hormonal level	No side effects
Combination of spearmint and flaxseed	Improves LH levels and promote folliculogenesis	No side effects
*M. arboreus*	Restores ovarian morphology and improves level of testosterone	No side effects
Marjoram extract	Improves PCOS symptoms	No side effects
Combination *Berberis integerrima* and resveratrol	Improves biochemical factor and regenerates ovarian morphology	No side effects
Combination of spinach and caraway	Improves biochemical factor and regenerates ovarian morphology	No side effects
*Vitex negundo* L.	Improves sex hormone levels and restores blood glucose and ovarian histology	No side effects
*P. muellerianus*	Improves blood glucose, lipid profile, and oxidative stress and prevents ovarian damage	No side effects
Cinnamon powder	Improves ovary morphology, level of testosterone, and insulin sensitivity	No side effects
*Labisia pumila* var. *Alata* (LPva)	Improves lipid profile and insulin resistance	No side effects

## Data Availability

Not applicable.
